# Product-Dependent Productivity Trends and Drought-Related GPP Anomalies in Planted and Natural Forests of Subtropical China

**DOI:** 10.3390/biology15212037

**Published:** 2026-11-08

**Authors:** Yanfeng Wang, He Li, Weidong Man, Mingyue Liu, Fengqin Yan, Pujia Yu, Feng Qian, Ying Liu, Zhi Ding

**Affiliations:** 1 Zhongke-Chongqing Research Institute of Big Data Technology and Application, Chongqing 401331, China; 2 School of Communication and Information Engineering, Chongqing University of Posts and Telecommunications, Chongqing 400065, China; 3 State Key Laboratory of Resources and Environmental Information System, Institute of Geographic Sciences and Natural Resources Research, Chinese Academy of Sciences, Beijing 100101, China; 4 College of Mining Engineering, North China University of Science and Technology, Tangshan 063210, China; 5 Chongqing Engineering Research Center for Remote Sensing Big Data Application, School of Geographical Sciences, Southwest University, Chongqing 400715, China; 6 National Field Observation and Research Station (Chongqing Jinfoshan) for Karst Ecosystem, School of Geographical Sciences, Southwest University, Chongqing 400715, China; 7 Key Laboratory of Reservoir Aquatic Environment, Chongqing Institute of Green and Intelligent Technology, Chinese Academy of Sciences, Chongqing 400714, China

**Keywords:** natural forest, planted forest, gross primary productivity, SPEI, drought exposure, post-drought anomaly

## Abstract

Natural and planted forests contribute substantially to regional gross primary productivity (GPP) in subtropical China, yet their long-term productivity trajectories and drought-related GPP responses remain uncertain, particularly across different satellite products. Using two satellite GPP products (MUSES and eLUE), meteorological records, and the Standardized Precipitation Evapotranspiration Index (SPEI), we compared long-term productivity trends in stable natural (796,468 km^2^) and planted forests (341,696 km^2^) and evaluated their responses during and after the 2016 summer drought relative to a 2001–2015 baseline. Both forest types experienced significant warming alongside sustained GPP increases; however, trend magnitudes differed across products, with eLUE indicating faster growth in planted forests and MUSES showing identical rates. Despite severe meteorological drought in summer 2016, both products indicated predominantly positive GPP anomalies, with SPEI explaining less than 1.5% of spatial anomaly variation. In the post-drought period (September–October), mean GPP anomalies declined across products, but the resulting status was highly product dependent: MUSES showed stronger negative anomaly changes in planted forests and below-baseline status, whereas eLUE indicated comparable anomaly changes between forest types and persistent above-baseline GPP. In conclusion, these findings highlight product-dependent interpretations of drought-related GPP responses, without establishing universal differences in drought resistance between forest types. These event-specific findings emphasize the need to distinguish meteorological drought exposure, short-term GPP anomaly changes, and productivity relative to historical seasonal baselines, without equating these indicators with long-term ecosystem resilience or recovery.

## Simple Summary

Natural and planted forests play important roles in absorbing carbon dioxide from the atmosphere, but their productivity and responses to drought may differ. This study compared long-term changes in forest productivity and responses to a major drought in 2016 across subtropical China using two satellite-based estimates of plant growth. Both forest types showed increasing productivity over time, but the estimated rates of increase differed between the two satellite products. During the drought, productivity was generally higher than the historical seasonal average. However, the products gave contrasting results for the following months: one indicated productivity below historical levels, particularly in planted forests, while the other indicated that productivity remained above historical levels. These findings highlight the uncertainty in satellite-based assessments of forest responses to drought. They also suggest that long-term productivity increases should not be interpreted as evidence of greater drought resistance. Considering differences between satellite products may improve forest monitoring and support more informed forest management and conservation decisions.

## 1. Introduction

Forests play a central role in terrestrial gross carbon assimilation and climate regulation [1]. However, forest productivity is spatially heterogeneous and may respond differently to changing climate across forest types. Natural and planted forests differ in species composition, stand structure, and management history, potentially leading to contrasting productivity dynamics and responses to climatic stress [2,3]. Distinguishing these forest types is therefore important for assessing long-term changes in gross primary productivity (GPP) and for interpreting ecosystem responses to climate extremes. In subtropical China, widespread afforestation and the persistence of natural forests provide an opportunity to compare forest types within a broadly shared monsoon-climate setting [3].

Warming may promote vegetation activity under temperature-limited conditions, whereas increased evaporative demand can intensify moisture constraints [4,5]. Recent field observations in subtropical China have demonstrated contrasting transpiration and evapotranspiration responses of evergreen and deciduous forests to compound heatwave–drought conditions [6]. Consequently, the productivity response to warming depends on the interaction between thermal and water availability conditions [7,8]. Elevated vapor pressure deficit can reduce stomatal conductance and photosynthetic rates even when soil moisture is not limiting [9,10]. The relationship between precipitation and vegetation productivity is also context dependent because water availability interacts with radiation, temperature, and other environmental constraints [8]. Therefore, long-term increases in GPP do not necessarily indicate reduced sensitivity to episodic drought. Examining long-term productivity trends together with responses to meteorological drought events is important for understanding productivity dynamics across contrasting timescales.

Drought reflects the combined effects of precipitation deficits and atmospheric evaporative demand and can affect forest productivity at spatial and temporal scales distinct from long-term climatic trends. The Standardized Precipitation Evapotranspiration Index (SPEI) provides a standardized measure of climatic water balance for characterizing drought exposure [11]. However, the extent to which meteorological drought severity corresponds to departures in GPP from its expected seasonal level remains uncertain, particularly across forest types. An additional uncertainty concerns how forest productivity should be interpreted following a drought event. Previous research has demonstrated persistent drought legacy effects in forest ecosystems [12], while recent studies have documented substantial heterogeneity in post-drought GPP responses across ecosystems and drought events [13,14]. However, changes in GPP anomalies between drought and subsequent observation periods do not necessarily indicate whether productivity has returned to its historical seasonal baseline. Assessing both the direction of anomaly change and the resulting post-drought anomaly status is therefore important for characterizing subsequent productivity dynamics without equating short-term changes with ecosystem recovery or resilience.

Differences between natural and planted forests may modify these relationships. Natural forests often contain more heterogeneous species assemblages and stand structures, whereas planted forests may exhibit greater uniformity in age or species composition [2,15]. Forest structural and compositional diversity can influence productivity and responses to climatic stress, as suggested by studies of mixed-species forests [16,17]. More directly, Ma et al. [18] reported higher drought risk in planted forests than in natural forests across China, based on resistance and resilience metrics. Yang et al. [19] found an opposite pattern in the Ili River Basin of Central Asia, where planted forests exhibited greater drought resistance but natural forests showed stronger post-drought resilience. These contrasting findings suggest that forest-type differences in drought responses depend on the study region, forest species, and the response metrics considered. However, drought resistance, resilience, and risk metrics are not equivalent to GPP anomalies or their spatial relationships with SPEI, and whether forest-type contrasts in GPP responses are consistent across different satellite productivity products remains uncertain. Furthermore, forest type is not an experimentally assigned treatment: differences in location, species, stand age, and management can confound comparisons [2,17]. Observed forest-type contrasts should therefore be interpreted as associations rather than direct evidence of a particular physiological mechanism or a universal advantage in drought resilience.

Satellite GPP products provide complementary but uncertain perspectives on these responses [13,14]. The eLUE product uses vegetation-index information within a light-use-efficiency framework [20], whereas MUSES incorporates additional radiation-related and meteorological stress constraints [21,22]. Crucially, these structural assumptions directly condition both long-term trend trajectories and short-term anomaly estimations. Because eLUE relies primarily on the Enhanced Vegetation Index (EVI) to quantify absorbed radiation, its long-term trend strongly mirrors canopy greening and leaf area expansion resulting from regional afforestation and warming-extended growing seasons. In contrast, MUSES explicitly differentiates sunlit and shaded leaves via a clearness-index weighting for diffuse radiation and applies dynamic temperature and atmospheric vapor pressure deficit (VPD) stress scalars. Under ongoing regional warming, intensified atmospheric evaporative demand and moisture stress can partially counter greening-induced photosynthetic gains in MUSES, potentially leading to more conservative multi-decadal GPP trends. Because their inputs and model structures differ, the products may lead to different interpretations of forest productivity responses to climatic extremes. Such discrepancies are particularly relevant when comparing natural and planted forests, because uncertainty in the estimated magnitude or direction of GPP anomalies may affect conclusions about their relative drought responses. Evaluating the consistency of forest-type contrasts across products is therefore important for distinguishing patterns supported by both products from product-dependent interpretations. However, whether sustained multi-decadal productivity increases confer tangible resilience to episodic moisture deficits, and the extent to which apparent differences between natural and planted forests stem from intrinsic ecological contrasts versus satellite model formulations, remains a critical knowledge gap. Here, we investigated long-term productivity trends and drought-related GPP responses in stable natural and planted forests across subtropical China using MUSES and eLUE. Drought events were identified using SPEI-based criteria, allowing productivity responses to be examined during and following a selected event. Specifically, we addressed three research questions: (1) How do long-term temperature, precipitation, and GPP trends differ between natural and planted forests? (2) Within drought-affected areas, how do drought-period GPP anomalies and their spatial relationships with drought severity differ between the two forest types? (3) How do GPP anomalies change from the drought to the post-drought period, and are forest-type differences in anomaly change and post-drought status consistent across GPP products?

## 2. Study Area

**Figure 1:**
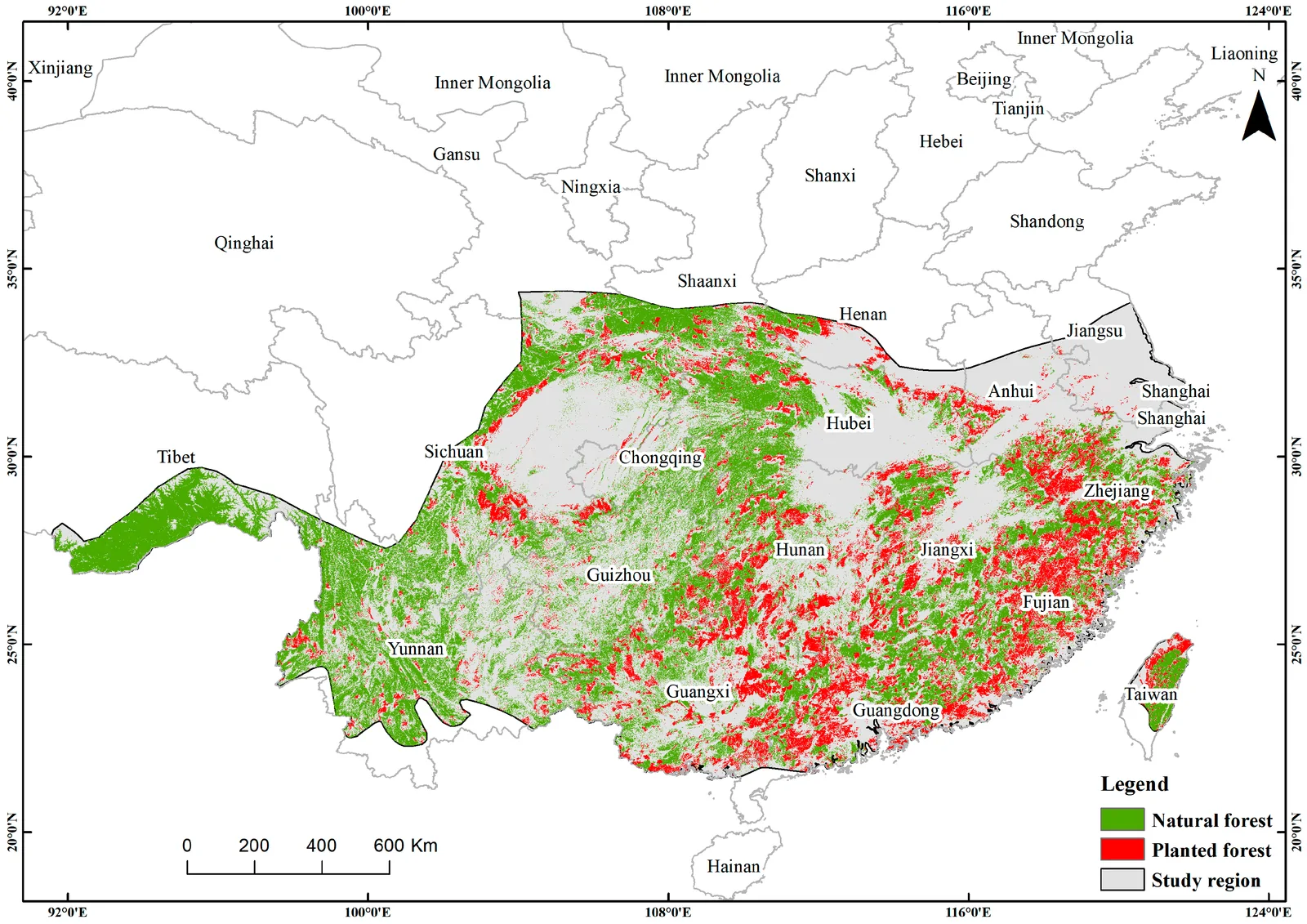
Study area.

The study area is located in the subtropical monsoon region of southern China (97°02′–122°07′ E, 21°23′–34°25′ N), extending southward from the Qinling Mountains–Huaihe River boundary and covering approximately 4.05 × 10^6^ km^2^ (Figure 1). The region is characterized by a humid subtropical monsoon climate, with pronounced seasonal variation in temperature and precipitation. Its climatic conditions are influenced by the East Asian monsoon and broader patterns of climate variability over China [23,24,25]. Seasonal precipitation and temperature variability create spatially heterogeneous moisture conditions and periodic drought exposure. The region includes natural evergreen broadleaf forests and extensive planted forests shaped by long-term land use and forest restoration programs, including the Natural Forest Protection Program and the Grain-for-Green Program [3,26,27]. Common planted species include Cunninghamia lanceolata, Pinus massoniana, and Eucalyptus spp. [3,28]. The coexistence of natural and planted forests across a climatically heterogeneous region provides a basis for comparing long-term GPP trends and drought-related productivity responses, while recognizing that forest types also differ in geographic and environmental context.

Natural and planted forest distributions were obtained from China’s Planted Forest Maps from 1990 to 2020, version 1.0 [28]. Details of the mapping framework and classification accuracy were reported by Cheng et al. [3]. These 30 m maps were developed using a Google Earth Engine-based framework combining multisource remote-sensing and ancillary features with field observations. Reported overall classification accuracies across the mapping years ranged from 77.3% to 81.8% [3]. We defined endpoint-consistent natural and planted forest areas as pixels assigned to the same forest category in both the 1990 and 2020 forest-type maps. For brevity, these areas are referred to as stable forests hereafter. This designation does not imply that forest type, stand composition, or management remained unchanged throughout the intervening period. The resulting mapped areas were 796,468 km^2^ for natural forests and 341,696 km^2^ for planted forests (Figure 1). The same forest-type classification was used to stratify climate and GPP analyses. The term “stable” refers only to agreement between the 1990 and 2020 forest-type maps; intermediate transitions cannot be excluded. As the forest maps have finite classification accuracy, both endpoint classification uncertainty and heterogeneity within a coarse GPP pixel may affect forest-type comparisons.

## 3. Data and Methods

### 3.1. Data

#### 3.1.1. Gross Primary Productivity (GPP) Data

Two satellite-derived gross primary productivity (GPP) products were used to assess long-term productivity and drought-related anomalies in natural and planted forests. The eLUE-GPP (MODIS) product applies an ecosystem light-use-efficiency model informed by the MODIS Enhanced Vegetation Index (EVI) and provides monthly global GPP estimates at 0.05° spatial resolution from February 2000 to March 2024 ([20]; https://doi.org/10.5061/dryad.v9s4mw74h). We analyzed the 2000–2020 period for long-term trends. The MUSES GPP product uses an improved MuSyQ-NPP-based light-use-efficiency approach that accounts for diffuse-radiation effects through a clearness-index weighting of sunlit and shaded leaves and applies temperature and water-stress constraints [21,22]. It incorporates GLASS vegetation products, meteorological reanalysis inputs, and MODIS land-cover information, and provides global estimates at 0.05° resolution and an 8-day interval for 1981–2018 (https://zenodo.org/records/3996814, accessed on 5 March 2026). We used MUSES data for 1990–2018. The 8-day GPP estimates were aggregated to monthly mean values by weighting each composite according to the number of days overlapping the corresponding calendar month. Monthly values were then used to construct annual mean daily GPP and the monthly climatological baseline. The two products differ in model formulation and temporal coverage; therefore, their long-term trends were evaluated over their respective available periods, whereas drought-related anomalies were assessed against the common 2001–2015 monthly reference period.

**Figure 2:**
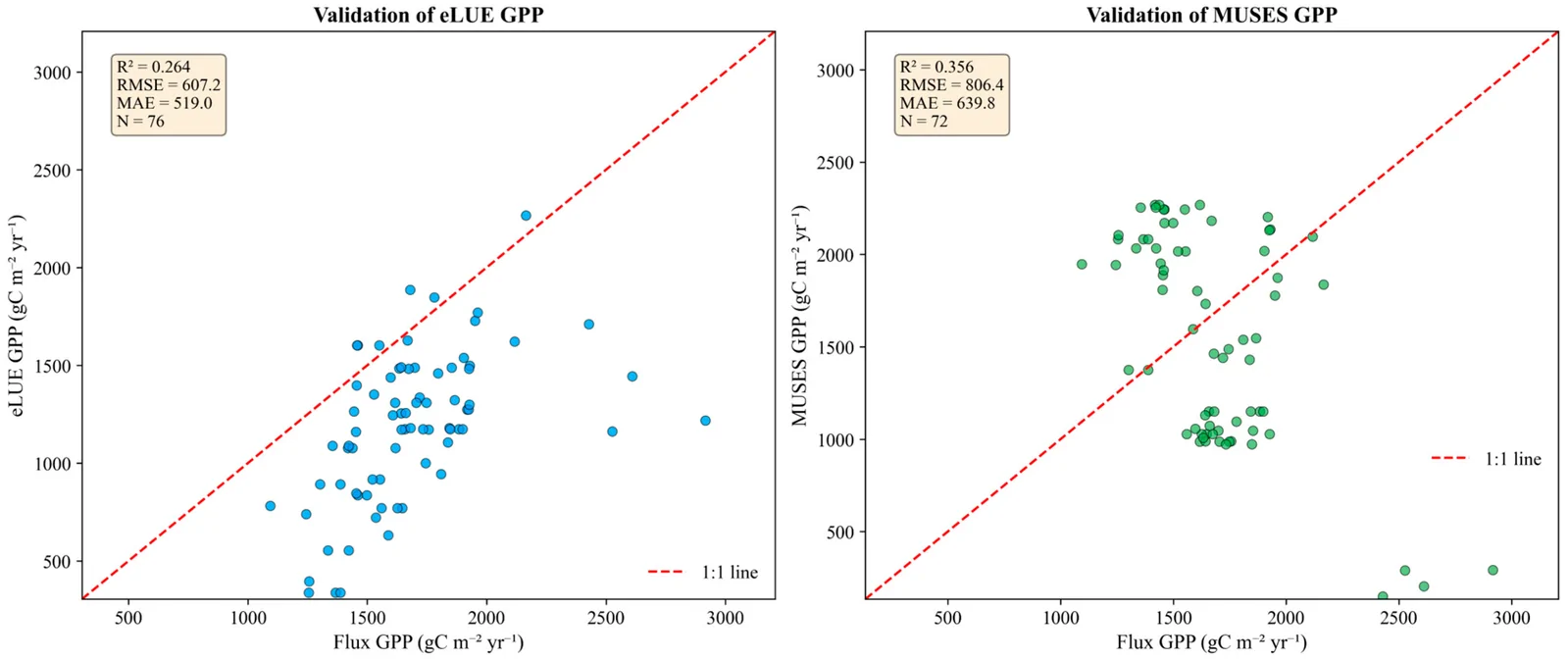
Validation of eLUE GPP and MUSES GPP by Flux data.

For an independent assessment of GPP product performance, annual satellite GPP estimates were compared with flux-tower-derived GPP records from the National Ecological Science Data Center (https://doi.org/10.57760/sciencedb.o00119.00077, accessed on 5 March 2026). The source dataset included 98 site-year observations from eight towers during 2003–2020; after exclusion of invalid records, 76 eLUE and 72 MUSES paired observations remained. The corresponding validation statistics were R^2^ = 0.264, RMSE = 607.2 g C m^−2^ yr^−1^, and MAE = 519.0 g C m^−2^ yr^−1^ for eLUE, and R^2^ = 0.356, RMSE = 806.4 g C m^−2^ yr^−1^, and MAE = 639.8 g C m^−2^ yr^−1^ for MUSES (Figure 2). These validation statistics describe the overall agreement between satellite-derived and tower-derived annual GPP. They do not independently validate forest-type-specific productivity anomalies during the selected 2016 drought event. Eddy-covariance systems observe net ecosystem CO_2_ exchange, from which site-level GPP is estimated by flux partitioning; the comparison here therefore uses flux-partitioning-derived GPP estimates rather than directly observed GPP. The reported annual-scale R^2^ and errors characterize imperfect agreement and do not establish the accuracy of monthly drought-period or post-drought anomalies. Under the conventional simple-linear-regression assumption that the site-year pairs are independent, the reported R^2^ values and sample sizes imply nominal *p*-values of approximately 2.1 × 10^−6^ for eLUE (*n* = 76, R^2^ = 0.264) and 3.2 × 10^−8^ for MUSES (*n* = 72, R^2^ = 0.356), calculated using F = [R^2^/(1 − R^2^)](*n* − 2), with 1 and *n* − 2 degrees of freedom. These *p*-values are derived from the published summary statistics and are not adjusted for repeated site-year observations within the eight towers. Thus, they should not be interpreted as independent-site significance or evidence that either product reliably captures monthly drought-related anomalies. Product-dependent responses are consequently treated as sensitivity of interpretation to GPP dataset selection rather than evidence that either product reproduces the event response more faithfully.

#### 3.1.2. Meteorological Data

Meteorological variables were obtained from the China Meteorological Forcing Dataset version 2.0 (CMFD 2.0; [29]). Monthly temperature and precipitation data for 1990–2020 were used to evaluate long-term climatic trends. For drought characterization, the meteorological input series covered 1980–2024, from which SPEI-3 was calculated. The CMFD 2.0 dataset provides near-surface meteorological variables at a spatial resolution of 0.1° and a temporal resolution of 3 h, covering the terrestrial area within 70° E–140° E and 15° N–55° N. This dataset integrates ERA5 reanalysis data (European Centre for Medium-Range Weather Forecasts) with meteorological station observations and incorporates AI-enhanced products (ISCCP-ITP-CNN and TPHiPr) for radiation and precipitation components, achieving significantly improved accuracy compared to its predecessor (CMFD 1.6; [30]). For this study, two key variables were extracted at the monthly scale: (1) near-surface (2 m) air temperature (unit: K), and (2) precipitation rate (unit: kg·m^−2^·s^−1^).

### 3.2. Methods

#### 3.2.1. Long-Term Trend Analysis

Temporal trends in gross primary productivity (GPP), air temperature, and precipitation were evaluated using the non-parametric Mann–Kendall (MK) test [31,32]. The MK test was applied to annual time series because it does not require the data to follow a normal distribution and is comparatively robust to outliers. A positive standardized Z statistic indicates an increasing trend, whereas a negative value indicates a decreasing trend. Trends were considered statistically significant at *p* < 0.05 (|Z| > 1.96). The magnitude of temporal change was quantified using the Theil-Sen slope estimator [33], defined as the median of all pairwise slopes in the time series.

Pixel-wise analyses were performed separately for stable natural-forest and planted-forest areas. Climatic trends were evaluated over 1990–2020. Long-term GPP trends were evaluated over the product-specific analysis periods, i.e., 1990–2018 for MUSES and 2000–2020 for eLUE.

#### 3.2.2. Drought Characterization and Definition of Drought Event

Drought conditions were characterized using the Standardized Precipitation-Evapotranspiration Index SPEI [11]. We calculated SPEI from monthly precipitation and air temperature data rather than using a precomputed SPEI product. The monthly climatic water balance was defined as:

*D_i_* = *P_i_ − PET_i_* (1)

where *P_i_* is monthly precipitation and *PET_i_* is potential evapotranspiration estimated using the Thornthwaite [34] method. The climatic water-balance series was accumulated over a three-month timescale and standardized to obtain SPEI-3 following Vicente-Serrano et al. [11]. Following conventional SPEI classifications, values ≤ −1.0, ≤−1.5, and ≤−2.0 were interpreted as moderate, severe, and extreme drought, respectively.

We examined monthly SPEI-3 during 1980–2024 to characterize the temporal evolution and spatial extent of meteorological drought across the study region (Figure S1). June–August 2016 was selected as the core drought period for the forest-type comparison because the analysis indicated extensive drought exposure during these months. The selection was based on the spatial distribution of SPEI and the temporal patterns shown in Figure S1; the event should not be interpreted as the longest or most severe drought across the full record without an explicit event-ranking analysis. September–October 2016 was defined as the subsequent observation window. This designation does not imply that meteorological drought had ended in every forest pixel. The 2016 episode was analyzed as a single, spatially extensive case study, not as a set of independent drought-event replicates. Winter–spring antecedent moisture, accumulated plant water stress, radiation, phenology, and soil-water availability were not incorporated as separate explanatory variables; their potential influence on June–October anomalies cannot be resolved. The term ‘post-drought’ denotes the subsequent September–October observation window and does not verify that drought ended at all sites.

For each spatial analysis pixel, drought exposure was characterized by the mean SPEI during June–August:

*SPEI_i_* = (*SPEI_i__,Jun_* + *SPEI_i__,Jul_* + *SPEI_i__,Aug_*)/3 (2)

where *SPEI_i_* represents the mean drought-period *SPEI* of pixel. A pixel was classified as drought affected when *SPEI_i_* ≤ −1.0. For mutually exclusive drought-severity classes, moderate drought was defined as −1.5 < *SPEI* ≤ −1.0, severe drought as −2.0 < *SPEI* ≤ −1.5, and extreme drought as SPEI ≤ −2.0. These classes were used to calculate the proportions of forest pixels in each drought-severity category.

#### 3.2.3. Quantifying Drought-Induced GPP Anomalies Using a Pixel-Specific Climatological Baseline

All drought-response analyses were restricted to stable forest areas that retained the same forest category between 1990 and 2020. The stable forest map had a spatial resolution of 1 km, whereas both GPP products had a spatial resolution of 5 km and the SPEI dataset had a spatial resolution of 10 km.

To preserve the original spatial information of the GPP products and avoid artificial replication of GPP observations through upsampling to a finer grid, all drought-response calculations were conducted on the 5-km grid of each GPP product. The 1-km stable forest-type map was mapped to each GPP grid using nearest-neighbor resampling because forest type is a categorical variable. Because categorical nearest-neighbor resampling does not quantify the proportion of each forest type within a coarse-resolution GPP pixel, mixed-pixel uncertainty may affect the estimated forest-type contrasts. The 10-km SPEI data were resampled to the corresponding 5-km GPP grid using bilinear interpolation because SPEI is a continuous variable. Natural and planted forest pixels were subsequently analyzed separately within each GPP product. Nearest-neighbor transfer assigns one forest category to each coarse GPP pixel without estimating natural- versus planted-forest fractional coverage. Thus, the label may not represent the entire coarse pixel. Bilinear interpolation of SPEI likewise does not provide stand-scale soil moisture or physiological water stress. These scale mismatches may attenuate or bias associations and should be considered when interpreting forest-type contrasts.

A pixel- and month-specific GPP climatology was constructed using 2001–2015 as the common baseline period for MUSES and eLUE. For each 5-km GPP pixel i and calendar month m, climatological GPP was calculated as:

*C_i_,_m_* = (1/15) Σ_*γ*=__2001_^2015^
*GPP_i_,_m_,ᵧ* (3)

where *C_i,m_* represents the historical mean GPP for the corresponding pixel and calendar month. Monthly GPP anomalies for 2016 were then calculated as:

*A_i_,_m_* = [(*GPP*_2016_*,_i_,_m_ − C_i_,_m_*)/*C_i_,_m_*] × 100 (4)

where *A_i,m_* is the GPP anomaly (%) of pixel *i* in month *m*. Calculations were restricted to pixels with valid GPP observations and positive climatological GPP values. This metric is expressed as a percentage. Negative values indicate productivity below the historical seasonal baseline, whereas positive values indicate productivity above the baseline. Because both positive and negative responses are possible, this metric is referred to as drought-period GPP change rather than GPP loss.

#### 3.2.4. Drought Exposure and Spatial SPEI–GPP Anomaly Relationships

Drought exposure was summarized separately for natural and planted forests using the mean and median June–August SPEI, the minimum monthly SPEI during June–August, and the proportions of forest pixels falling below the moderate, severe, and extreme drought thresholds. Monthly GPP anomalies were subsequently summarized for drought-affected forest pixels to characterize the temporal evolution of productivity before, during, and after the core drought period.

To characterize the spatial relationship between meteorological drought severity and GPP anomalies, separate ordinary least-squares regressions were fitted for natural and planted forests within each GPP product. For pixel i, the predictor variable was the mean June–August SPEI:

*S_i_* = *mean*(*SPEI_i__,Jun_*, *SPEI_i_,_Jul_*, *SPEI_i_,_Aug_*) (5)

and the response variable was the mean June–August GPP anomaly:

*A_i_* = *mean*(*A_i_,_Jun_*, *A_i_,_Jul_*, *A_i_,_Aug_*) (6)

Separate ordinary least-squares regressions were fitted for natural and planted forests within each GPP product:

*A_i_* = *α* + *βS_i_* + *ε_i_* (7)

For each regression, the slope (β), intercept, Pearson correlation coefficient (*r*), coefficient of determination (R^2^), slope standard error, and *p* value were calculated. Because lower SPEI values indicate stronger drought, a positive slope implies increasingly negative GPP anomalies toward more severe drought conditions, whereas a negative slope indicates the opposite response along the observed drought gradient. Importantly, these regressions characterize spatial associations between drought-period SPEI and GPP anomalies among pixels meeting the drought criterion. They do not estimate causal drought effects or intrinsic physiological sensitivity, and their conventional significance tests do not account for spatial dependence.

To test whether the spatial SPEI–GPP anomaly relationships differed between forest types, a forest-type-by-SPEI interaction model was fitted:

*A_i_* = *β*_0_ + *β*_1_*S_i_* + *β*_2_*F_i_* + *β*_3_(*S_i_ × F_i_*) + *ε_i_* (8)

where *F_i_* was coded as 0 for natural forest and 1 for planted forest. The interaction coefficient *β_3_* tests whether the slope of the SPEI-GPP anomaly relationship differs between natural and planted forests. The model was fitted independently for MUSES and eLUE. The regression and group-comparison tests were performed at the pixel level without explicit adjustment for spatial autocorrelation. Accordingly, the resulting *p* values represent conventional pixel-level significance and should be interpreted cautiously. Because nearby pixels share climatic conditions and may not be statistically independent, conventional pixel-level *p* values should not be interpreted as evidence from independent drought events or as spatially corrected inference. Particular emphasis is placed on effect direction and the very small R^2^ rather than on *p* values alone.

#### 3.2.5. Drought-Period GPP Response and Post-Drought Dynamics

Drought-period GPP anomalies were calculated as the arithmetic mean of the monthly percentage anomalies during June–August 2016. This definition was used consistently in the analyses of drought-period productivity, SPEI–GPP anomaly relationships, and subsequent changes in GPP anomalies.

Post-drought dynamics were evaluated for September-October 2016. First, mean post-drought GPP anomaly was calculated for each pixel:

*A_post_i_* = *mean*(*A_i_,_Sep_*, *A_i_,_Oct_*) (9)

The change in GPP anomaly from the drought period to the post-drought period was then calculated as:

Δ*A_post_i_* = *A_post_i_ − A_drought_i_* (10)

where *A_drought_i_* is the mean June–August GPP anomaly. Because Δ*A_post_i_* represents the difference between two percentage anomalies, it is expressed in percentage points (pp). Positive values indicate that GPP anomaly increased after the drought period, whereas negative values indicate a further decline relative to the drought-period anomaly.

In addition, post-drought GPP status was represented by *A_post_i_* itself (%). Values close to zero indicate productivity near the corresponding historical seasonal baseline, negative values indicate productivity remaining below the baseline, and positive values indicate productivity exceeding the historical baseline. These two metrics describe changes in a satellite-estimated ecosystem process, not direct evidence of individual-tree resistance, recovery, drought resilience, or legacy effects. A September–October anomaly is not a measure of recovery duration, and positive anomalies during drought do not imply that drought enhanced GPP.

#### 3.2.6. Statistical Comparison and Spatial Outputs

Forest-type differences in drought-period GPP change, post-drought GPP change, and post-drought GPP status were evaluated separately for each GPP product using two-sided Mann–Whitney U tests. Because pixel-level distributions were not assumed to be normally distributed, central tendency and dispersion were summarized primarily using the median and interquartile range (25th–75th percentiles), with means also retained for descriptive comparison. Statistical tests were conducted only for forest pixels satisfying the drought criterion (mean June–August SPEI ≤ −1.0).

Spatial outputs were generated on the native grid of each GPP product. The drought-period mean SPEI raster was masked to all stable forest pixels so that the full spatial gradient of drought exposure within forests remained visible. In contrast, GPP-response rasters—including drought-period GPP anomaly, drought-period GPP change, post-drought GPP change, and post-drought GPP status—were masked to stable forest pixels that also satisfied the drought criterion. All non-forest pixels and forest pixels outside the defined affected area were assigned No Data in the corresponding GPP-response rasters.

## 4. Results

### 4.1. Climatic Background and Long-Term GPP Dynamics of Natural and Planted Forests

**Figure 3:**
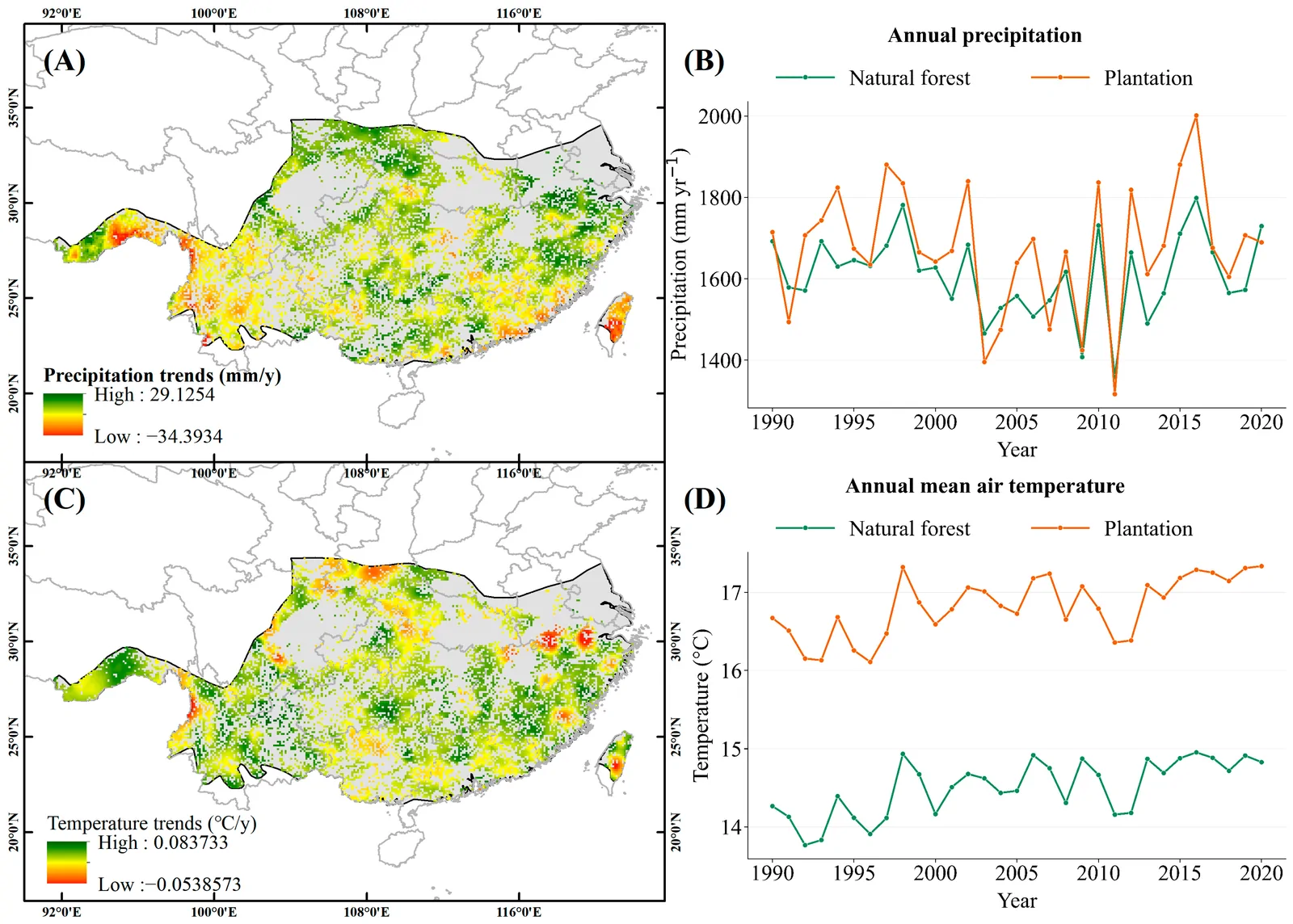
Spatial and temporal variations in precipitation and air temperature in natural and planted forests across subtropical China during 1990–2020. (**A**) Spatial distribution of annual precipitation trends. (**B**) Interannual variations in mean annual precipitation in natural and planted forests. (**C**) Spatial distribution of annual mean air temperature trends. (**D**) Interannual variations in mean annual air temperature in natural and planted forests. Precipitation and temperature trends are expressed in mm yr^−1^ and °C yr^−1^, respectively.

**Figure 4:**
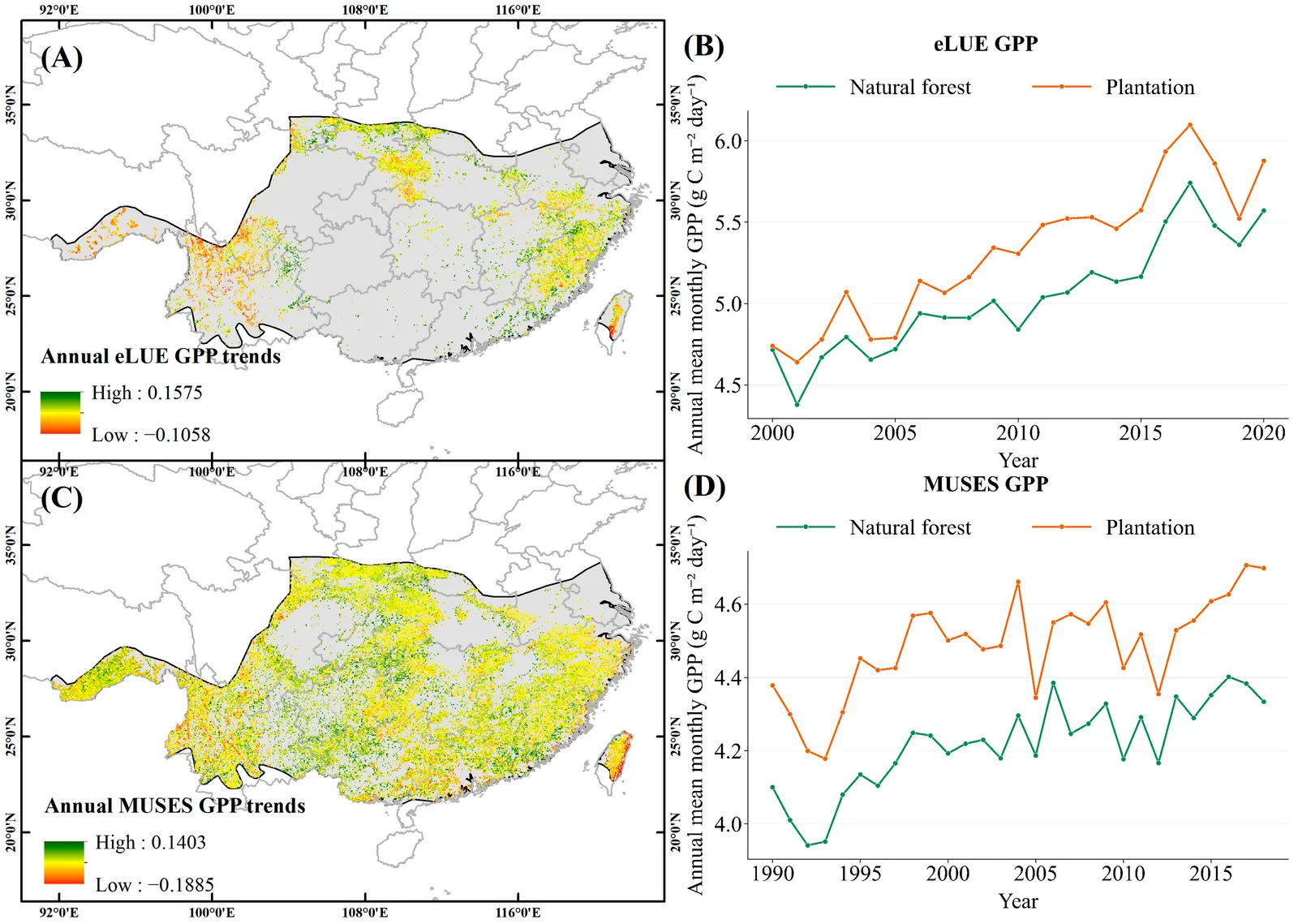
Spatial and temporal patterns of long-term gross primary productivity (GPP) trends in natural and planted forests across subtropical China derived from eLUE and MUSES products. (**A**) Spatial distribution of GPP trends derived from eLUE during 2000–2020. (**B**) Interannual variations in mean daily GPP in natural and planted forests derived from eLUE during 2000–2020. (**C**) Spatial distribution of GPP trends derived from MUSES during 1990–2018. (**D**) Interannual variations in mean daily GPP in natural and planted forests derived from MUSES during 1990–2018. GPP trends are expressed in g C m^−2^ day^−1^ yr^−1^, and mean daily GPP is expressed in g C m^−2^ day^−1^.

Annual precipitation exhibited strong interannual fluctuations during 1990–2020 but no significant regional trend in either natural (−1.00 mm yr^−1^) or planted forests (−0.33 mm yr^−1^; Figure 3B, Table 1). Spatially, localized drying was mainly confined to coastal and southern margins (Figure 3A, Table 1). In contrast, regional warming was pervasive and statistically significant in both forest types (~0.026–0.029 °C yr^−1^, *p* < 0.001), with over 75% of pixels experiencing significant temperature increases (Figure 3C,D; Table 1). Both GPP products indicated sustained, significant productivity increases, though their estimated growth rates and forest-type contrasts differed (Figure 4, Table 1). MUSES estimated identical regional GPP growth rates for natural and planted forests (0.011 g C m^−2^ day^−1^ yr^−1^; Figure 4D), whereas eLUE indicated a faster increase in planted forests than in natural stands (0.066 vs. 0.049 g C m^−2^ day^−1^ yr^−1^; Figure 4B). Across both products, positive GPP trends predominated spatially (covering 38–83% of forest pixels), and planted forests consistently maintained higher regional mean daily productivity than natural forests (Figure 4, Table 1).

### 4.2. Drought Exposure and Spatial Relationships Between SPEI and GPP Anomalies

**Figure 5:**
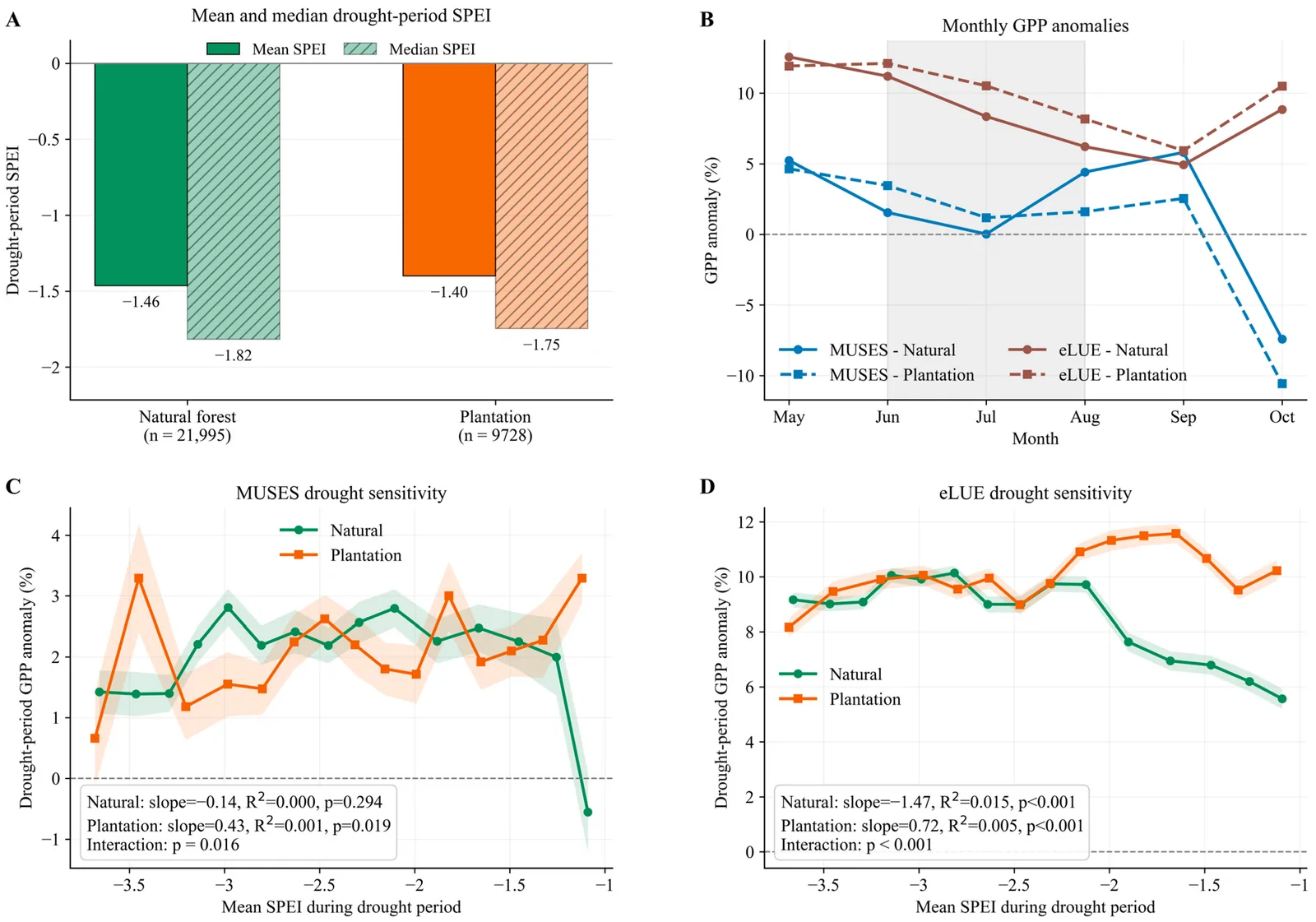
Meteorological drought exposure and drought-related GPP anomalies in natural and planted forests during the 2016 summer drought in subtropical China. (**A**) Mean and median SPEI during June–August 2016 for natural and planted forests. (**B**) Monthly GPP anomalies derived from MUSES and eLUE in natural and planted forests from May to October 2016. (**C**) Spatial relationship between mean drought-period SPEI and GPP anomalies derived from MUSES. (**D**) Spatial relationship between mean drought-period SPEI and GPP anomalies derived from eLUE. Drought-period SPEI and GPP anomalies in panels (**C**) and (**D**) represent mean values during June–August 2016. GPP anomalies are expressed as percentage deviations from the corresponding 2001–2015 monthly climatology. Lower SPEI values indicate more severe meteorological drought.

**Figure 6:**
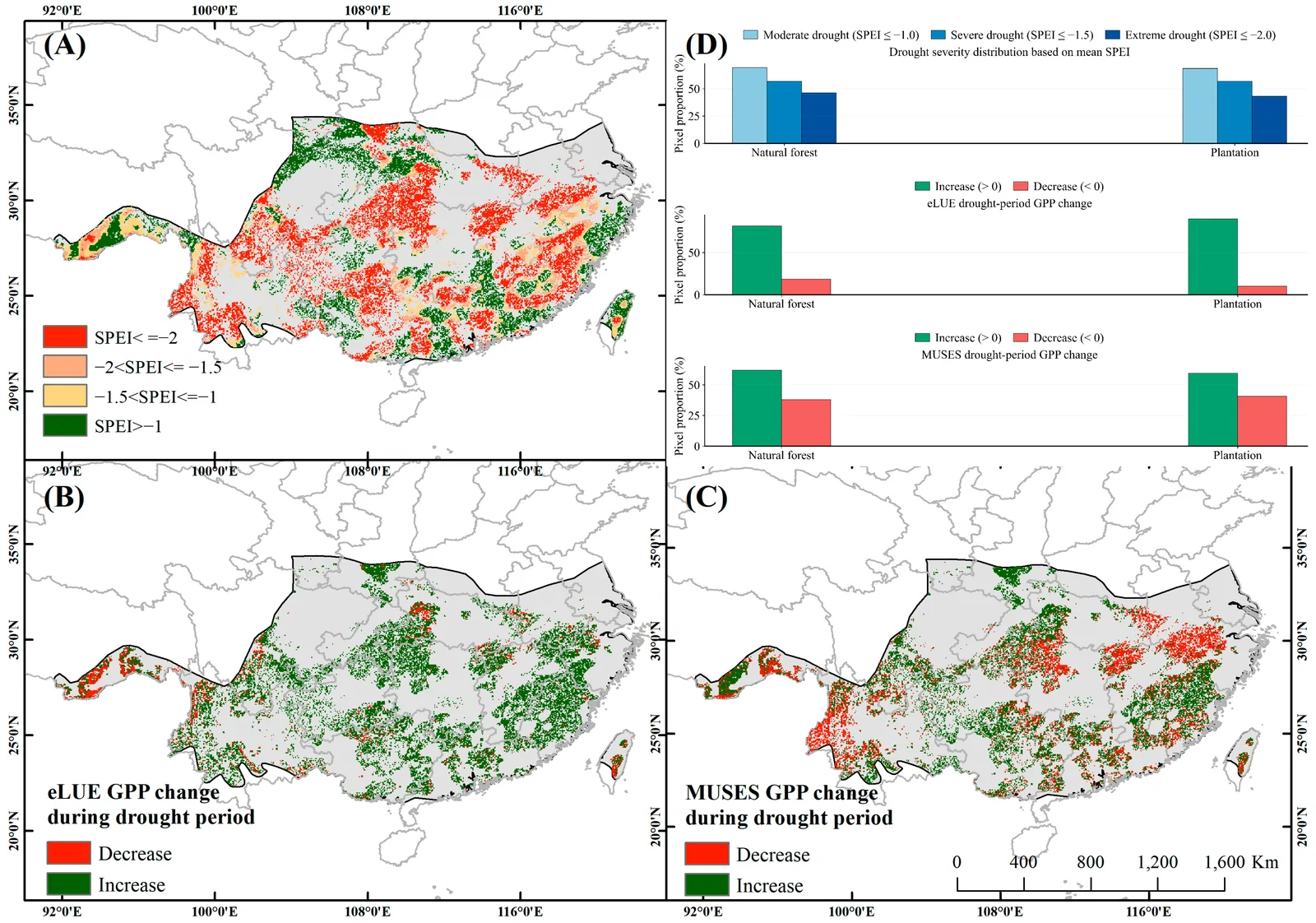
Spatial patterns of meteorological drought exposure and drought-period GPP anomalies in natural and planted forests across subtropical China during summer 2016. (**A**) Spatial distribution of mean SPEI during June–August 2016. (**B**) Spatial distribution of positive and negative drought-period GPP anomalies derived from eLUE. (**C**) Spatial distribution of positive and negative drought-period GPP anomalies derived from MUSES. (**D**) Proportions of natural- and planted-forest pixels across drought-severity classes and the proportions exhibiting positive or negative GPP anomalies derived from eLUE and MUSES. Drought-affected pixels were defined by mean June–August SPEI ≤ −1.0. Positive and negative GPP anomalies indicate productivity above and below the corresponding 2001–2015 monthly climatology, respectively. GPP anomaly maps in panels (**B**) and (**C**) are restricted to drought-affected forest pixels.

The 2016 summer drought imposed severe and comparable climatic stress across both forest types, with mean SPEI reaching approximately −1.4 (median < −1.7) and extreme drought affecting over 40% of their total areas (Figure 5A and Figure 6A,D). Despite widespread water deficits, both satellite products revealed predominantly positive GPP anomalies relative to the 2001–2015 climatology during June–August, with positive departures exceeding 60% of pixels in MUSES and 75% in eLUE (Figure 5B and Figure 6B–D). Spatial relationships between drought severity (SPEI) and GPP anomalies differed significantly between forest types (interaction *p* < 0.05; Table 2). Planted forests exhibited positive slopes in both products (i.e., smaller GPP anomalies under more severe drought), whereas natural forests showed a flat (MUSES) or negative relationship (eLUE; Figure 5C,D; Table 2). Crucially, however, SPEI accounted for less than 1.5% of the spatial variance in GPP anomalies across all regressions (R^2^ ≤ 0.0146), demonstrating that meteorological drought intensity alone provides limited power to predict spatial productivity departures (Table 2).

### 4.3. Post-Drought GPP Anomaly Dynamics in Natural and Planted Forests

**Figure 7:**
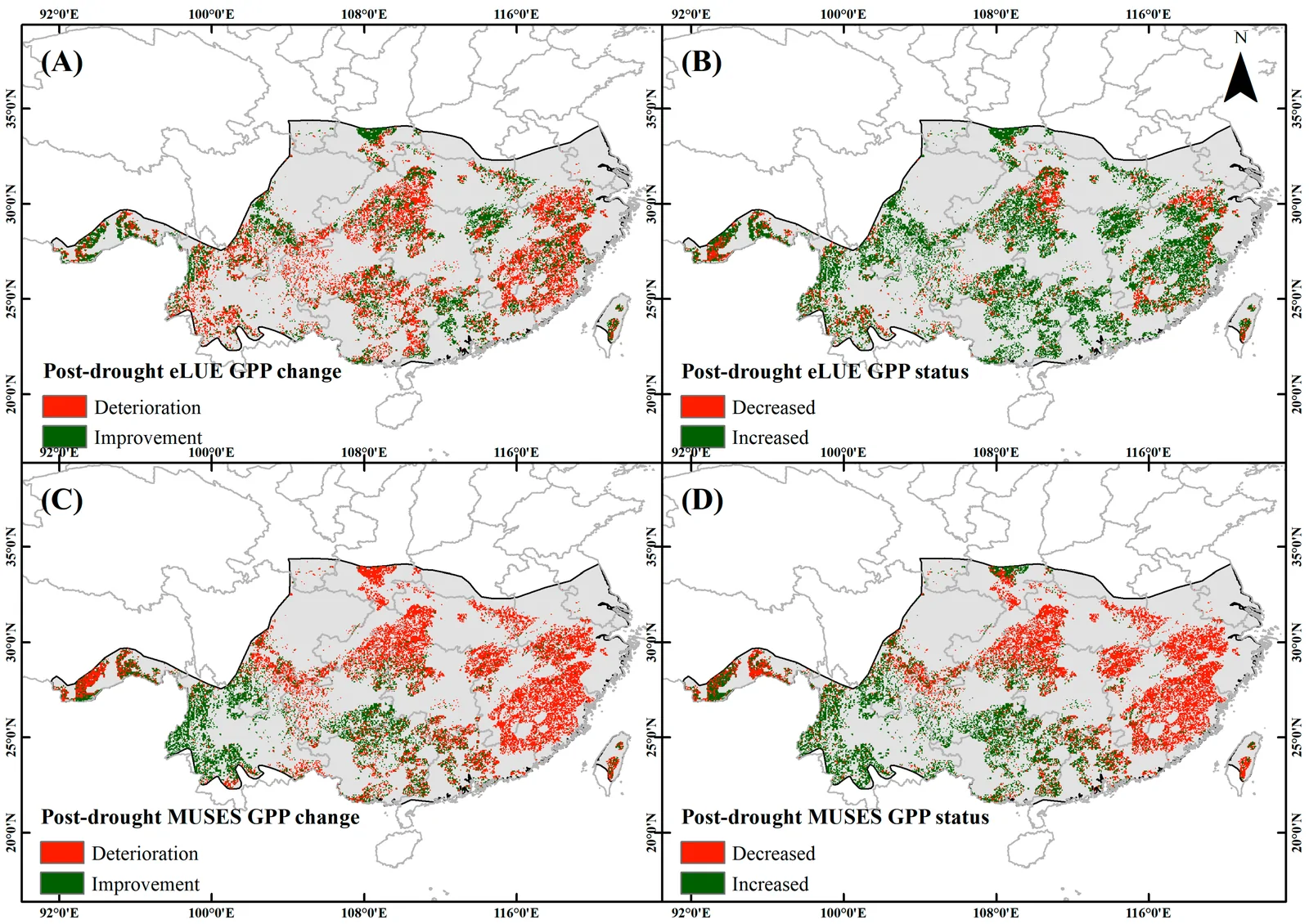
Spatial patterns of post-drought GPP anomalies and anomaly changes. (**A**) Post-drought GPP anomaly change derived from eLUE. (**B**) Post-drought GPP anomaly derived from eLUE. (**C**) Post-drought GPP anomaly change derived from MUSES. (**D**) Post-drought GPP anomaly derived from MUSES. GPP anomaly change represents the difference between post-drought (September–October) and drought-period (June–August) mean GPP anomalies, expressed in percentage points (pp). Post-drought GPP anomaly represents the percentage deviation from the corresponding 2001–2015 monthly climatology. Positive and negative anomaly changes indicate improvement and deterioration relative to the drought period, respectively, whereas positive and negative anomalies indicate GPP above and below the historical baseline, respectively.

**Figure 8:**
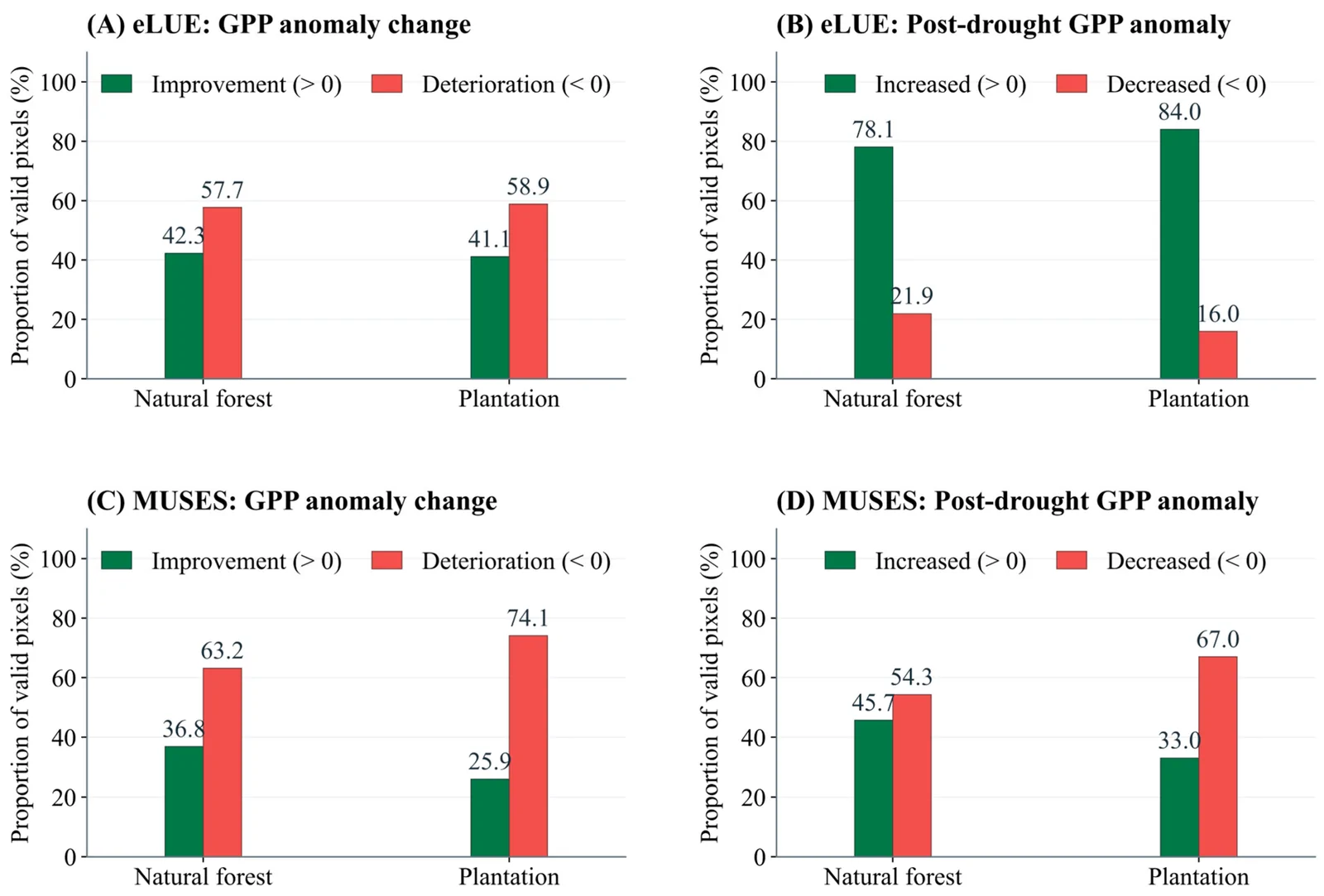
Proportions of forest pixels with positive and negative post-drought GPP responses in natural and planted forests. (**A**) Post-drought GPP anomaly change derived from eLUE. (**B**) Post-drought GPP anomaly derived from eLUE. (**C**) Post-drought GPP anomaly change derived from MUSES. (**D**) Post-drought GPP anomaly derived from MUSES. Positive and negative anomaly changes indicate improvement and deterioration relative to the drought period, respectively, whereas positive and negative post-drought anomalies indicate GPP above and below the historical baseline, respectively. Proportions were calculated relative to the total number of valid pixels for each forest type and GPP product.

**Figure 9:**
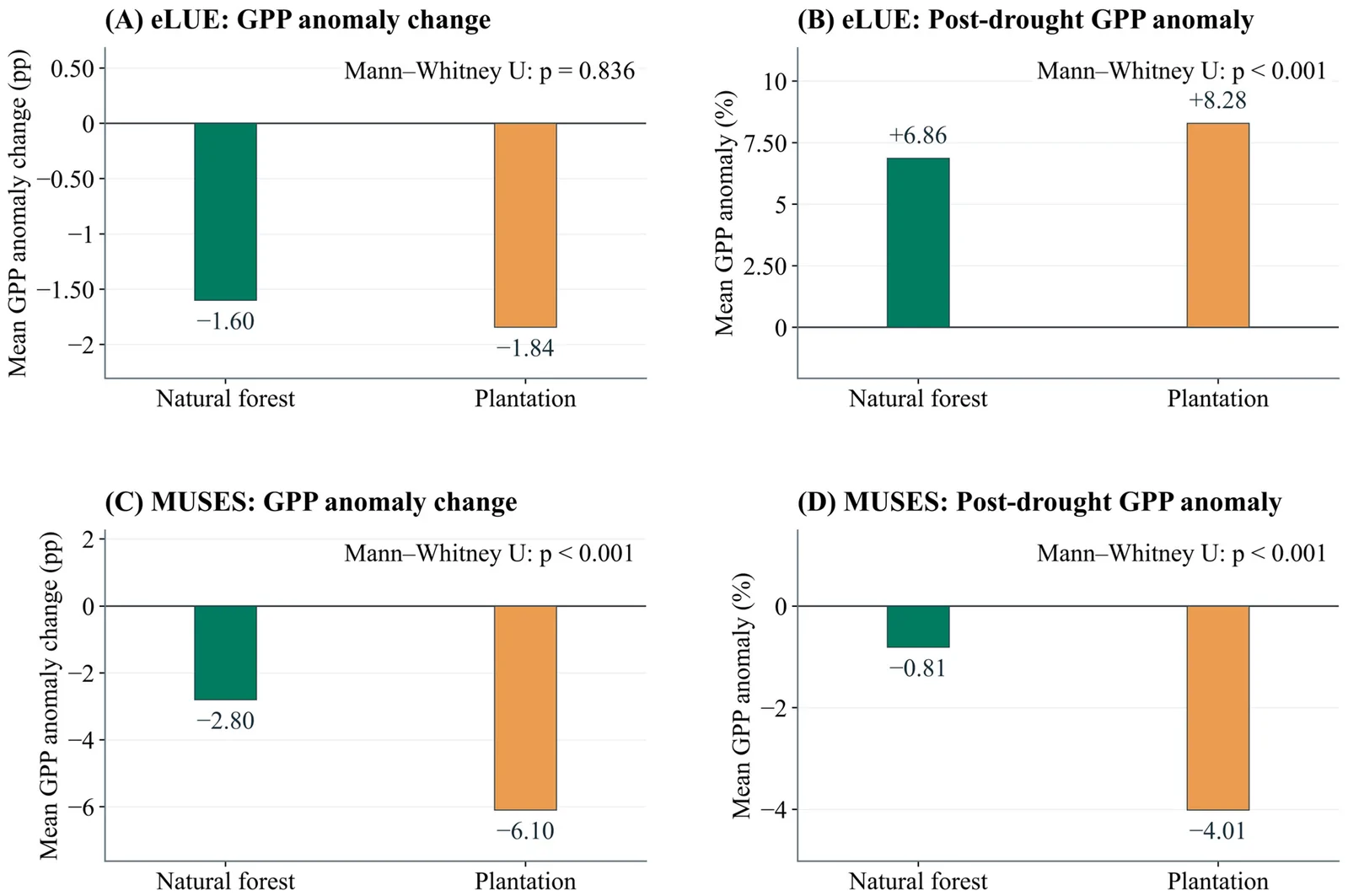
Differences in post-drought GPP anomalies and anomaly changes between natural and planted forests. (**A**) Post-drought GPP anomaly change derived from eLUE. (**B**) Post-drought GPP anomaly derived from eLUE. (**C**) Post-drought GPP anomaly change derived from MUSES. (**D**) Post-drought GPP anomaly derived from MUSES. Bars represent the mean values for each forest type. The horizontal dashed line indicates zero, separating improvement from deterioration in anomaly change and positive from negative post-drought GPP anomalies. Differences between natural and planted forests were assessed using the Mann–Whitney U test, with corresponding *p* values shown in each panel.

From the core drought period (June–August) to the post-drought window (September–October), both products indicated widespread declines in GPP anomalies, with negative anomaly changes (ΔA_post < 0) occurring across 57–74% of forest pixels (Figure 7A,C and Figure 8A,C). Spatially, this post-drought deterioration was concentrated mainly across the central and eastern portions of the study region (Figure 7A,C). Despite consistent declining trajectories, the post-drought productivity status and forest-type contrasts diverged substantially between products (Figure 8 and Figure 9). For eLUE, anomaly declines were modest and did not differ significantly between forest types (−1.60 pp in natural vs. −1.84 pp in planted forests; Mann–Whitney U test, *p* = 0.836), with over 78% of pixels maintaining positive anomalies relative to the seasonal baseline (Figure 8B and Figure 9A,B). Conversely, MUSES indicated significantly stronger anomaly deterioration in planted forests than in natural stands (−6.10 pp vs. −2.80 pp, *p* < 0.001), resulting in predominantly below-baseline productivity (mean post-drought anomalies of −4.01% and −0.81%, respectively; Figure 8D and Figure 9C,D). Thus, while both products captured a general post-drought reduction in productivity anomalies, the inferred absolute status and forest-type sensitivity depended heavily on product choice.

## 5. Discussion

This study examined three linked dimensions of forest productivity in subtropical China: long-term trends, responses during a SPEI-identified drought event, and changes following that event. Both natural and planted forests exhibited increasing GPP against a background of regional warming, yet this long-term increase did not translate into a simple ranking of drought performance. During the selected 2016 summer event, extensive meteorological drought co-occurred with predominantly positive GPP anomalies in both products, while spatial SPEI–GPP associations differed between forest types but explained little variation. After the drought period, anomalies generally declined, although the resulting productivity status and forest-type contrasts depended strongly on the GPP product. These findings highlight why long-term trends, drought exposure, drought-period anomalies, and post-drought dynamics should be interpreted as distinct dimensions rather than interchangeable measures of resilience.

### 5.1. Long-Term Warming and Product-Dependent GPP Trends

Both forest types experienced significant warming during 1990–2020 without significant regional precipitation trends, while MUSES and eLUE each indicated increasing GPP. This combination suggests that forest productivity increased under a changing climatic background, but the temporal coincidence of warming and rising GPP does not establish a causal relationship. Temperature may influence phenology and photosynthetic activity, whereas changes in atmospheric CO_2_, radiation, forest age, species composition, and management may also contribute [4,5]. Specifically, nationwide ecological restoration projects and elevated atmospheric CO_2_ have been identified as primary drivers of long-term vegetation greening and gross productivity enhancement across southern China [35,36]. Because the present analysis quantified trends rather than partitioning their drivers, the relative contributions of these processes remain unresolved.

The forest-type comparison further cautions against inferring a universal productivity advantage from trend rates. MUSES estimated the same regional GPP trend for natural and planted forests (0.011 g C m^−2^ day^−1^ per year), whereas eLUE estimated a larger trend in planted forests (0.066 versus 0.049 g C m^−2^ day^−1^ per year). The products cover different periods (1990–2018 and 2000–2020), and their model formulations also differ; consequently, their trend magnitudes cannot be treated as a controlled, same-period comparison, and differences in their estimated trends cannot be attributed solely to differences in product algorithms. Agreement that productivity increased is more defensible than a product-independent claim about which forest type increased faster. A common-period comparison would be needed to distinguish temporal-coverage effects from algorithm-related differences.

Importantly, higher annual GPP or a stronger positive long-term trend does not, by itself, imply greater resistance to an episodic moisture deficit. Long-term productivity integrates multiple favorable and unfavorable conditions, whereas drought-period anomalies represent departures from a seasonal reference. Treating these measures separately provides the necessary context for interpreting the event-based results without assuming a productivity–drought-resilience trade-off in advance.

### 5.2. Extensive Drought Exposure but Limited Explanatory Power of SPEI for GPP Anomalies

The selected 2016 summer event affected both forest types extensively, with similar average drought-period SPEI and widespread extreme-drought exposure. Nevertheless, both MUSES and eLUE estimated predominantly positive GPP anomalies relative to the 2001–2015 monthly climatology. This apparent contrast is not logically inconsistent: SPEI measures climatic water-balance anomalies, whereas GPP anomalies measure deviations from a product-specific estimate of normal seasonal productivity. A negative SPEI value therefore does not require a negative GPP anomaly. Moreover, positive anomalies during drought should not be interpreted as evidence that drought increased photosynthesis; they describe the estimated state relative to the chosen baseline, not the counterfactual effect of drought.

Several processes could contribute to the weak correspondence between meteorological drought exposure and GPP anomalies, including soil-water storage, rooting conditions, radiation availability, and antecedent climatic conditions. In humid subtropical regions, reduced cloud cover during meteorological droughts often enhances incident solar radiation, which can temporarily stimulate photosynthetic carbon assimilation before soil moisture drops below critical thresholds [37,38]. Furthermore, deep-root water uptake and access to groundwater reservoirs can effectively buffer forest canopies against atmospheric evaporative demand during short-term precipitation deficits [39]. In humid regions, reduced cloudiness during relatively dry conditions may also increase incoming radiation and potentially favor photosynthetic activity [8]. However, these explanations were not directly tested in the present study. The coexistence of positive and negative GPP responses across the drought-affected region, together with differences between MUSES and eLUE, indicates that regional mean anomalies can conceal substantial spatial heterogeneity. Consequently, the observed positive regional signal should not be generalized to all forest stands or interpreted as an absence of drought stress.

The significant forest-type × SPEI interactions in MUSES (*p* = 0.016) and eLUE (*p* < 0.001) suggest that the spatial associations between drought severity and GPP anomalies differed between natural and planted forests. In planted forests, positive slopes imply lower anomalies at more negative SPEI values, whereas eLUE estimated an opposite-sign association in natural forests. Yet the regressions explained at most 1.46% of spatial variation (R^2^ ≤ 0.0146). Thus, statistical significance, potentially amplified by large pixel counts and spatial dependence, should not be equated with strong predictive performance or intrinsic physiological sensitivity. Differences in forest location, stand characteristics, and environmental conditions may also contribute to these associations.

These results address the second research question by showing that similar regional drought exposure does not necessarily yield the same spatial GPP response, but they do not identify a causal mechanism for the forest-type contrasts. Spatially explicit models that account for autocorrelation and include soil moisture, vapor pressure deficit, radiation, stand composition, and antecedent conditions would be needed to determine whether the contrasting slopes persist after environmental differences are controlled.

Previous studies have reported contrasting patterns of drought resistance and post-drought resilience between natural and planted forests. Ma et al. [18] found higher drought risk in planted forests across China, whereas Yang et al. [19] reported greater drought resistance in planted forests but stronger post-drought resilience in natural forests in the Ili River Basin of Central Asia. Moreover, the present analysis focuses on subtropical monsoon forests rather than arid and semi-arid ecosystems, further limiting direct comparability. From a functional perspective, forest diversity can influence drought responses, although its protective effects vary among forest types and environmental contexts [40]. Hydraulic trait diversity may buffer ecosystem flux responses under drought [41]. Comparative evidence from dryland forests also indicates that planted and natural forests may differ in drought vulnerability, with the outcome influenced by environmental and soil conditions [42].

### 5.3. Post-Drought Anomaly Dynamics and Divergence Between GPP Products

Post-drought responses illustrate why anomaly change and anomaly status should be assessed jointly. The former measures the difference between September–October and June–August anomalies (percentage points), whereas the latter measures September–October productivity relative to the historical seasonal baseline (%). Both products indicated predominantly negative anomaly changes after the drought period, but this deterioration did not necessarily imply below-normal GPP. In eLUE, approximately 58% of pixels in each forest type deteriorated, while positive post-drought anomalies still occurred in 78.1% of natural-forest and 84.0% of planted-forest pixels. A reduction in a previously positive anomaly can therefore coexist with above-baseline productivity. Recent studies have shown that drought-related GPP effects can persist beyond the drought period and vary substantially among ecosystems [13,14]. Our results complement these findings by demonstrating that short-term post-drought anomaly status can also differ markedly between satellite GPP products. However, the present two-month observation window does not allow the duration or mechanisms of drought legacy effects to be determined.

The product contrast was stronger for the post-drought anomaly status than for the general direction of change. MUSES showed deterioration in 63.2% of natural-forest and 74.1% of planted-forest pixels and negative post-drought anomalies in 54.3% and 67.0%, respectively. Mean anomaly changes were also more negative in planted forests (−6.10 pp) than in natural forests (−2.80 pp), with a significant difference between their distributions. By contrast, eLUE estimated similar mean anomaly changes (−1.84 and −1.60 pp in planted and natural forests), and the Mann–Whitney U test did not detect a distributional difference (*p* = 0.836). Although eLUE showed a significant forest-type difference in post-drought anomaly distributions, both forest types retained positive mean anomalies (+8.28% and +6.86%, respectively). Hence, the conclusion that plantations experienced a distinctly stronger post-drought deterioration is supported by MUSES but is not reproduced consistently by eLUE.

Differences in vegetation inputs, stress constraints, and model formulation may help explain the divergence between MUSES and eLUE [20,21,22]. MUSES incorporates explicit temperature and water stress scalars and accounts for diffuse radiation effects through a clearness-index weighting, whereas eLUE relies on vegetation-index information within a light-use-efficiency framework. These structural differences may lead to different sensitivities to drought-induced physiological downregulation versus canopy structural change. Satellite greenness indices (such as the EVI used in eLUE) typically reflect canopy structural properties and chlorophyll retention, which often lag behind immediate stomatal closure and physiological stress during and immediately following drought [43,44,45]. Consequently, greenness-based models tend to underestimate the immediate severity of drought-induced photosynthetic decline compared to models that explicitly integrate meteorological stress scalars [43]. Nevertheless, the present comparison cannot isolate which model component caused the discrepancy or establish that one product more accurately captured physiological drought stress. Existing flux-tower evaluations do not constitute independent verification of forest-type-specific responses during the selected event. The short September–October window also precludes conclusions about the duration or completeness of recovery. Event-period eddy-covariance observations, complementary vegetation-function indicators, and a longer post-drought series would be needed to determine whether the product-specific patterns represent biological differences, retrieval uncertainty, or both.

The annual tower-based evaluation cannot adjudicate which product more accurately captured 2016 month-specific anomalies. Disagreement may reflect model inputs, environmental stress representations, and temporal differences, but those candidate explanations were not isolated in the present analysis. No product is designated as the ground truth for this event.

### 5.4. Implications for Forest Assessment and Management

For forest assessment, the most defensible implication is that productivity level, response during drought, and subsequent anomaly status should be reported separately. Both forest types exhibited long-term GPP increases, but the selected event did not provide a product-independent ranking of drought resistance or recovery. In particular, the stronger negative post-drought changes estimated for planted forests by MUSES were not confirmed by eLUE. These findings therefore do not demonstrate a general productivity–stability trade-off, nor do they establish that natural forests recover faster. Management interpretations based solely on a single GPP product or a single response metric would risk overstating the evidence.

Monitoring programs could instead combine long-term productivity indicators with drought exposure, spatially resolved anomaly changes, and post-drought anomaly status. Such information may help identify locations where productivity remains below its historical seasonal baseline or where anomalies are deteriorating, while acknowledging uncertainty between products. Conservation of natural forests and diversification of planted stands may offer broader ecological benefits [2], but this analysis did not test the effectiveness of specific management practices for drought mitigation. Management recommendations about forest-type resilience require additional information on species, stand age, site conditions, and repeated drought events.

Finally, GPP should not be equated with net carbon sequestration. It represents gross photosynthetic uptake and excludes respiratory losses, disturbance emissions, and changes in carbon stocks. Neither higher GPP nor a positive post-drought anomaly establishes a more persistent net carbon sink. The present findings are therefore most appropriately framed as differences in estimated productivity dynamics, rather than definitive evidence about carbon-sink stability. Specifically, gross photosynthetic CO_2_ fixation is not interchangeable with net ecosystem CO_2_ exchange, net ecosystem productivity, or net carbon storage; the latter require accounting for respiratory and other carbon fluxes.

### 5.5. Limitations and Future Directions

Several limitations affect interpretation. First, the stable-forest mask was based on forest classifications at the endpoints of the analysis period, which does not establish unchanged species composition, age, management, or even uninterrupted class membership throughout the intervening years. Resampling categorical forest data to the coarser GPP grids may also introduce mixed-pixel and classification uncertainty. Second, MUSES and eLUE differ in algorithm and temporal coverage; product comparisons of long-term trends are not based on identical observation periods, and available site-level evaluations do not independently validate forest-type-specific GPP anomalies during the selected drought event. This case study did not control for pre-event hydroclimatic history, local soil and groundwater conditions, species identity, stand age, or management, nor did it assess repeated drought events. Consequently, forest-type differences may combine multiple confounded environmental and stand-level influences; a coarse categorical forest label is not an experimentally isolated forest-origin effect. The comparison is explicitly descriptive and event-specific: forest-type labels are not experimental treatments, and the observed differences cannot be generalized to all natural or planted forests or attributed uniquely to forest origin. Previous droughts and winter–spring hydroclimatic conditions may have altered soil-water reserves, canopy phenology, plant hydraulic status, and subsequent GPP; because these antecedent histories were not evaluated separately, the present analysis cannot distinguish current-event associations from legacy or carry-over effects. Likewise, stand age, species composition, soils, groundwater access, and management may contribute to within-type heterogeneity and between-type contrasts. Importantly, September–October 2016 is a subsequent observation window rather than a verified period of complete drought cessation or ecological recovery. The two-month window cannot establish whether GPP returned to its pre-event level, the time required for any recovery, or the persistence of delayed effects. We therefore do not rank the two forest types by drought resilience or recovery rate; our reported changes and statuses are short-term, product-dependent GPP anomalies relative to calendar-month baselines.

Third, the drought-period SPEI–GPP regressions had very low explanatory power and did not explicitly address spatial autocorrelation or environmental confounding. Reported pixel-level *p* values may therefore overstate inferential precision. Fourth, the analysis focused on one selected event and a September–October post-drought window. Although month-specific historical baselines reduce seasonal comparability problems, changing radiation, temperature, and other conditions can still influence anomaly differences between periods. The design cannot resolve delayed effects, recovery duration, or whether the observed contrasts recur across events of different intensity, timing, and duration.

Future studies should compare GPP products over common years, use spatially robust statistical approaches, and examine multiple independently selected drought events with longer follow-up periods. Independent observations during and after drought, together with soil moisture, atmospheric demand, stand composition, age, and management data, would help evaluate product accuracy and separate forest-type associations from environmental effects. Linking GPP anomalies with net ecosystem exchange and biomass observations could further assess whether short-term productivity dynamics translate into longer-term carbon-balance consequences.

## 6. Conclusions

This study compared long-term gross primary productivity (GPP) trends and drought-related GPP anomalies in natural and planted forests across subtropical China using two satellite-derived GPP products (MUSES and eLUE). Both forest types exhibited increasing GPP over their respective observation periods, although the estimated trend magnitudes and forest-type contrasts differed between products. During the 2016 summer drought, both products indicated predominantly positive GPP anomalies relative to the historical monthly baseline, while meteorological drought severity explained less than 1.5% of the spatial variation in these anomalies. Importantly, these findings describe a specific drought event and do not establish a universal advantage in drought sensitivity or resistance for either forest type.

Both GPP products indicated a decline in productivity anomalies from June–August to September–October 2016, but their estimates of subsequent productivity status and forest-type differences diverged. MUSES suggested stronger negative anomaly changes in planted forests and below-baseline productivity, whereas eLUE indicated similar anomaly changes between forest types and generally above-baseline productivity. This product dependence highlights inherent uncertainties in satellite-derived assessments of short-term forest productivity responses, without establishing which product more accurately represents ecosystem functioning during or following the selected drought event.

Several limitations constrain the interpretation and generalizability of these findings. The analysis focused on a single drought event, and differences between natural and planted forests may reflect variations in species composition, stand age, soil properties, groundwater availability, management practices, and other environmental conditions rather than forest origin alone. Antecedent hydroclimatic conditions and previous drought events were not explicitly evaluated, preventing the separation of current-event responses from potential carry-over effects. Furthermore, the two-month September–October observation window does not verify the complete cessation of drought, whether productivity returned to its pre-event state, or the duration of subsequent recovery. Accordingly, the observed forest-type contrasts should not be generalized beyond the study conditions or interpreted as evidence of differences in long-term ecosystem drought resilience or recovery rates.

The principal contribution of this study is to decouple meteorological drought exposure, changes in GPP anomalies between observation periods, and productivity status relative to a historical seasonal baseline, while explicitly considering uncertainty associated with satellite product selection. Future research should examine multiple independent drought events with longer observation periods, incorporate antecedent hydroclimatic conditions and forest structural characteristics, and integrate satellite estimates with independent field-based measurements. Such approaches would help determine the consistency of forest-type differences across environmental conditions and improve assessments of long-term ecosystem responses to drought.

## Figures and Tables

**Table 1:** Long-term trends in climate variables and gross primary productivity (GPP) in natural and planted forests.

Variable	Period	Forest Type	Regional Slope	MK Z	MK *p*	Mean Pixel Slope	Median Pixel Slope	Significant Increase (%)	Significant Decrease (%)
Precipitation	1990–2020	Natural	−1.00	−0.48	0.634	−1.45	−0.67	2.1	9.1
		Plantation	−0.33	−0.07	0.946	−0.40	0.08	1.6	1.9
Temperature		Natural	0.026	3.84	<0.001	0.027	0.028	75.9	0.8
		Plantation	0.029	3.88	<0.001	0.028	0.029	80.0	0.5
MUSES GPP	1990–2018	Natural	0.011	4.82	<0.001	0.012	0.011	42.7	3.9
		Plantation	0.011	4.03	<0.001	0.010	0.010	38.5	3.9
eLUE GPP	2000–2020	Natural	0.049	5.04	<0.001	0.044	0.044	65.5	0.6
		Plantation	0.066	5.22	<0.001	0.055	0.054	82.5	0.3

Note: Slope represents the Theil–Sen trend of the annual mean time series for each forest type. MK Z and MK *p* denote the test statistic and *p* value of the Mann–Kendall trend test, respectively. Mean pixel slope and median pixel slope represent the mean and median of pixel-level Theil–Sen slopes. Significant increase and significant decrease indicate the percentages of valid pixels exhibiting positive and negative trends, respectively (*p* < 0.05). Precipitation and temperature trends are expressed in mm yr^−1^ and °C yr^−1^, respectively. GPP is expressed as annual mean daily productivity (g C m^−2^ day^−1^), and the reported GPP slopes indicate the annual rate of change in this quantity.

**Table 2:** Relationships between drought-period SPEI and GPP anomalies in natural and planted forests.

GPP Product	Forest Type	n	Slope (β)	R^2^	*p*	Interaction *p*
MUSES	Natural forest	15,260	−0.136	0.0001	0.294	0.016
MUSES	Plantation	6667	0.432	0.0008	0.019	
eLUE	Natural forest	14,699	−1.468	0.0146	<0.001	<0.001
eLUE	Plantation	6577	0.723	0.0055	<0.001	

Note: β represents the regression slope of GPP anomaly (%) against drought-period mean SPEI. R^2^ is the coefficient of determination, and *p* indicates the statistical significance of the regression. The interaction *p* value indicates the significance of the difference in regression slopes between natural and planted forests.

## Data Availability

The datasets used in this study are publicly available from their respective repositories. China’s Planted Forest Maps are available at https://www.nesdc.org.cn/sdo/detail?id=672d67777e28174998e6324a (accessed on 5 March 2026). The eLUE-GPP product is available at https://doi.org/10.5061/dryad.v9s4mw74h. The MUSES GPP product is available at https://zenodo.org/records/3996814 (accessed on 5 March 2026). CMFD data are available at https://data.tpdc.ac.cn/en/data/e60dfd96-5fd8-493f-beae-e8e5d24dece4 (accessed on 5 March 2026). SPEI-3 was calculated by the authors from the meteorological inputs described in Section 3.2.2 and was not obtained as a precomputed drought-index product.

## Supplementary Materials

The following supporting information can be downloaded at: https://www.mdpi.com/article/10.3390/biology15212037/s1, Figure S1: Temporal patterns of meteorological drought in the study region during 1980–2024. (**a**) Monthly regional mean SPEI and its linear trend; (**b**) annual proportion of drought-affected area; and (**c**) annual proportion of drought months. Gray shading highlights 2016, the year selected for detailed analysis of forest GPP responses.
