# Supplementary material for: Product-Dependent Productivity Trends and Drought-Related GPP Anomalies in Planted and Natural Forests of Subtropical China
Source: Biology (Basel). 2026 Nov 8;15(21):2037. doi: 10.3390/biology15212037 (PMC13762095; doi:10.3390/biology15212037)
Supplement: Supplementary file 1 [file biology-15-02037-s001.zip › biology-4666867-supplementary.pdf]

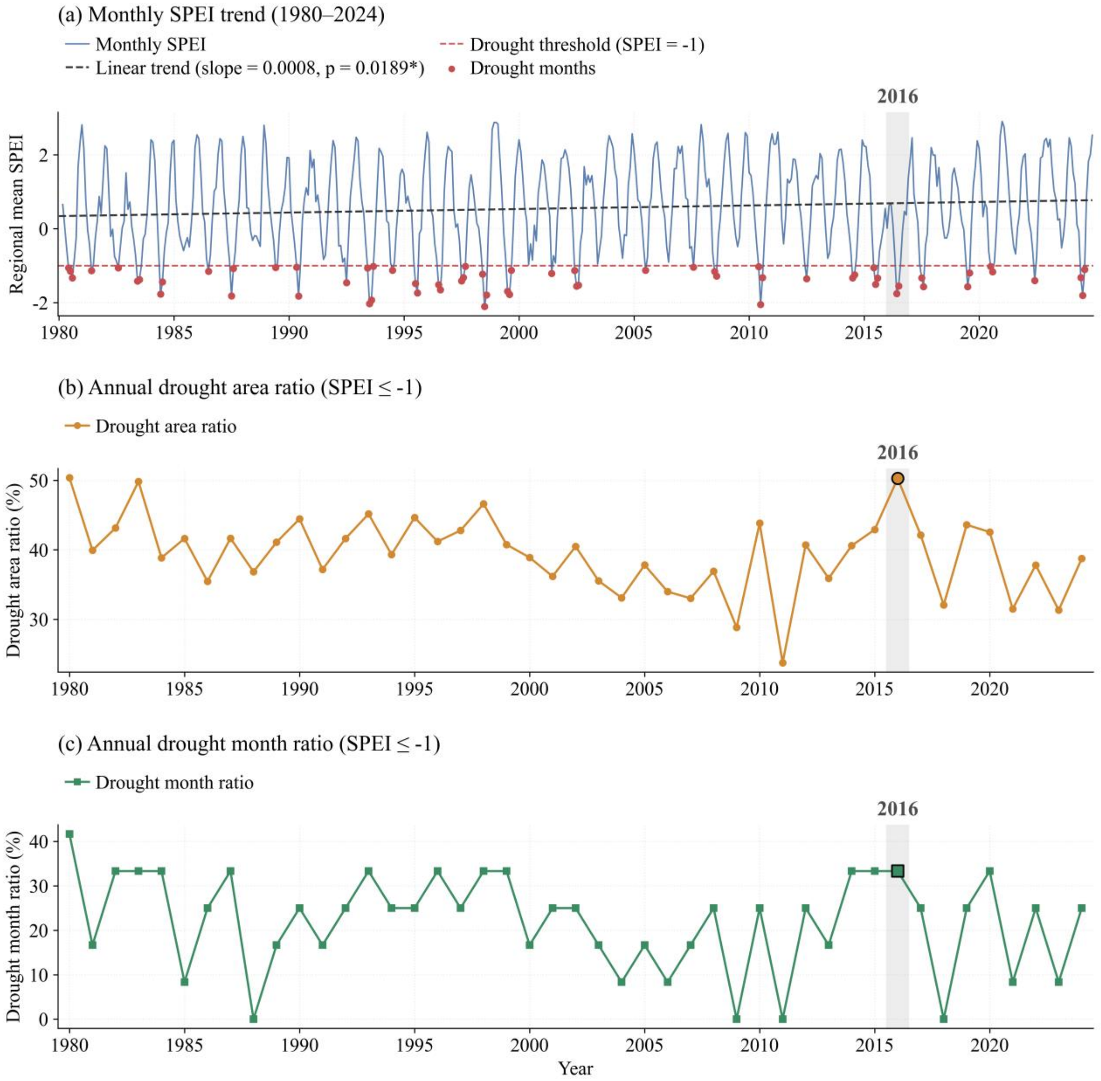

Figure S1 Temporal patterns of meteorological drought in the study region during 1980–2024. (a) Monthly regional mean SPEI and its linear trend; (b) annual proportion of drought-affected area; and (c) annual proportion of drought months. Gray shading highlights 2016, the year selected for detailed analysis of forest GPP responses.
